# Perceived Public Stigma Toward Psychological Help: Psychometric Validation of the Stigma Scale for Receiving Psychological Help Among Chinese Law Students

**DOI:** 10.3390/bs15081084

**Published:** 2025-08-10

**Authors:** Tingting Wang, Qi Lu Huang, Wei Li

**Affiliations:** 1 School of Civil and Commercial Law, Shandong University of Political Science and Law, Jinan 250014, China; wangtingting@sdupsl.edu.cn; 2 Department of Social and Behavioural Sciences, City University of Hong Kong, Hong Kong, China; qlhuang5-c@my.cityu.edu.hk; 3 Law School, Central University of Finance and Economics, Beijing 100081, China

**Keywords:** public stigma, help-seeking attitudes, Chinese, law students, scale validation

## Abstract

Public stigma toward psychological help-seeking is a critical barrier to mental health service utilization, particularly among university students in culturally conservative and academically demanding disciplines such as law. In China, where Confucian values emphasize social conformity and face preservation, law students may internalize societal narratives that associate mental illness with personal weakness, deterring them from accessing psychological services. This study translated and examined the psychometric properties of the Stigma Scale for Receiving Psychological Help (SSRPH) among Chinese law students. A total of 1257 undergraduate law students from five universities in China participated in the study. Exploratory factor analysis (EFA) was conducted on a randomly selected subsample (*n* = 628) to examine the scale’s factor structure, followed by confirmatory factor analysis (CFA) on a second subsample (*n* = 629). Results supported a unidimensional factor structure with strong internal consistency (*α* = 0.82). CFA yielded a good model fit (CFI = 0.97, TLI = 0.95). Significant negative correlations with help-seeking attitudes (*r* = −0.37, *p* < 0.001) supported discriminant validity. While further validation is warranted, the Chinese SSRPH appears suitable for assessing perceived public stigma in legal education contexts and may inform future research and program design in stigma reduction and mental health promotion.

## 1. Introduction

Mental health concerns represent a growing public health priority worldwide. According to recent estimates, one in eight individuals globally experiences a mental disorder, with depression and anxiety ranking among the most common conditions (World Health Organization, 2022). In China, the prevalence of mental disorders is similarly significant. A nationally representative survey found that 16.6% of adults had experienced a diagnosable mental illness in their lifetime (Huang et al., 2019). Despite the growing demand for mental health support in China, service utilization remains remarkably low. Numerous studies have documented that over 80% of individuals diagnosed with a mental disorder in China do not seek professional psychological assistance, regardless of their education level or employment status (Gearing et al., 2024; Shi et al., 2020). This striking treatment gap cannot be solely attributed to service availability or resource constraints. Rather, a growing body of research identifies stigma as a persistent and culturally embedded barrier to help-seeking (Chen, 2018; Shi et al., 2020).

Stigma related to mental health is a multifaceted phenomenon shaped by cultural, institutional, and interpersonal dynamics. Corrigan and Watson (2002) distinguish between public stigma, which reflects widespread societal attitudes toward people with mental illness, and self-stigma, which occurs when individuals internalize these negative beliefs. Public stigma often involves stereotypes that portray people with mental illness as dangerous, weak, or incompetent. These stereotypes can provoke fear, anger, and rejection, ultimately resulting in discriminatory behaviors, such as social exclusion or unequal access to education and employment (Corrigan & Rao, 2012; Yanos et al., 2015). When internalized, such attitudes can erode self-esteem and reduce motivation to seek treatment, further reinforcing cycles of psychological distress and marginalization (Vogel et al., 2006; Büchter & Messer, 2017; Dubreucq et al., 2021).

In collectivist cultures such as China, stigma is further reinforced by cultural norms that emphasize family reputation, obedience, and emotional restraint. A key sociocultural construct in this context is the concept of “face” (面子, *mianzi*), which refers to an individual’s social image and perceived standing within the community. Seeking psychological help may be perceived as a sign of weakness or failure, thereby resulting in “loss of face” (失面子) for both the individual and their family. In other words, disclosing a mental health issue may be interpreted as a personal and familial failing, thus jeopardizing one’s social standing and perceived competence (Yin et al., 2020). Several studies have documented that concerns about losing face are strongly associated with reluctance to seek help (Cheang & Davis, 2014; Parker et al., 2001). For Chinese university students, especially those enrolled in competitive academic disciplines, the social costs of seeking psychological support may outweigh the perceived benefits, resulting in continued underutilization of available resources.

Law students represent a particularly vulnerable subgroup. Legal education in China is characterized by highly structured curricula, pressure to excel in national judicial examinations, and expectations of professional perfectionism. These students are often socialized into values, such as rationality, composure, and individual competence, which may be at odds with acknowledging emotional distress (Ferris, 2021; Lan et al., 2024). While international studies have consistently reported high levels of psychological distress among law students (Organ et al., 2016), few investigations have focused on Chinese law students specifically. The demands of legal training, compounded by the stigma of help-seeking, may create an environment where mental health issues are both common and concealed.

Several psychological theories provide a framework for understanding these dynamics. The Theory of Planned Behavior (Ajzen, 1991) proposes that behavioral intentions are shaped by attitudes, subjective norms, and perceived behavioral control. Stigma influences all three components by fostering negative attitudes toward psychological services, promoting norms that discourage disclosure, and reducing perceived control over seeking help (Mackenzie et al., 2004; Shek et al., 2022). Corrigan’s Social Cognitive Model further explains how public stigma can lead to self-stigma, undermining personal agency and motivation (Corrigan & Watson, 2002). From the perspective of the Conservation of Resources theory (Hobfoll, 2001), individuals facing significant stress may avoid help-seeking behaviors to prevent loss of valuable resources, such as academic reputation or social standing. For law students, these combined theoretical perspectives suggest that stigma is not only a personal barrier but also a culturally and structurally reinforced deterrent.

Despite the central role of stigma in shaping help-seeking behavior, few culturally appropriate tools are available to measure it within Chinese academic populations. The Stigma Scale for Receiving Psychological Help (SSRPH), developed by Komiya et al. (2000), is a widely used scale for assessing perceived public stigma. While brief and easy to administer, prior research has raised concerns regarding its dimensionality, cultural sensitivity, and construct clarity (Tucker et al., 2013; Pinto et al., 2015). In Chinese contexts, certain terminology, such as “seeking professional psychological help”, may carry different connotations, shaped by local understandings of mental health, status hierarchies, and institutional trust (Zhou et al., 2019). Cross-cultural applications of the SSRPH have shown inconsistent psychometric performance, highlighting the need for population-specific validation (Vogel et al., 2017; Zhou et al., 2019).

At present, no studies have systematically validated the SSRPH for use among Chinese law students. Given the social expectations, cultural stigma, and academic pressures unique to this group, such an effort is both timely and necessary. Without such tools, it is difficult to meaningfully assess stigma or evaluate interventions aimed at promoting psychological well-being within legal education settings.

### The Present Study

This study addresses the critical gap by translating and validating the SSRPH for use among Chinese law students, a population underrepresented in stigma research despite their high psychological vulnerability. Building on prior literature, the study evaluates the scale’s factorial structure, internal consistency, and discriminant validity in a large multi-university sample. By providing a psychometrically sound and culturally sensitive instrument, the study contributes both theoretically and practically to the literature on mental health stigma, with implications for assessment, policy, and targeted intervention in high-pressure academic environments.

## 2. Materials and Methods

### 2.1. Participants and Procedure

Participants were 1257 undergraduate law students (mean age = 20.57 years, *SD* = 1.12, 45.51% female) recruited from five comprehensive universities located in diverse regions of China, Beijing (North), Shanghai (East), Guangzhou, Guangdong Province (South), Wuhan, Hubei Province (Central), and Xuzhou, Jiangsu Province (East), to ensure geographical and cultural diversity. Inclusion criteria required that participants (a) be currently enrolled in an undergraduate law program and (b) provide informed consent. No exclusion criteria were based on gender, year of study, or prior mental health experience.

Data collection was conducted in two phases between March and May 2023 using a stratified sampling method. Each university disseminated the survey via its official student communication platforms. After reading and agreeing to the informed consent statement, participants completed an anonymous online questionnaire, hosted on the Qualtrics survey platform. The survey took approximately 15 min to complete. The study protocol was approved by the Ethics Review Committee of the School of Civil and Commercial Law, Shandong University of Political Science and Law.

To allow for independent validation, the total sample was randomly divided into two subsamples. Sample A (*n* = 628) was used for exploratory factor analysis (EFA), and Sample B (*n* = 629) was used for confirmatory factor analysis (CFA).

### 2.2. Measures

#### 2.2.1. Stigma Scale for Receiving Psychological Help (SSRPH)

The SSRPH, originally developed by Komiya et al. (2000), is a five-item self-report measure assessing perceived public stigma toward seeking psychological help. Each item is rated on a 4-point Likert scale (0 = strongly disagree to 3 = strongly agree). Higher scores indicate greater perceived stigma, with good internal consistency in prior research (e.g., α = 0.80; Logsdon et al., 2009). The Chinese version used in this study was translated through a forward-backward translation process and reviewed by a bilingual panel of experts to ensure conceptual and cultural equivalence. Minor wording adjustments were made to enhance clarity and relevance for Chinese university students.

#### 2.2.2. Attitudes Toward Seeking Professional Psychological Help–Short Form (ATSPPH-SF)

To assess discriminant validity, participants completed the ATSPPH-SF (Fischer & Farina, 1995), a 10-item measure of general attitudes toward psychological help-seeking. Responses are rated on a 4-point Likert scale (0 = disagree to 3 = agree), with higher scores reflecting more positive attitudes toward help-seeking. The scale has been demonstrated to have good internal consistency in prior research (Cronbach’s *α* = 0.77 to 0.83; e.g., Elhai et al., 2008).

#### 2.2.3. Sociodemographic Characteristics

Participants completed a brief self-report questionnaire that assessed basic sociodemographic information, including age, gender, and current enrollment status as a law student in China. Respondents who were not currently enrolled in a law program were directed to the end of the survey and were not included in the study.

### 2.3. Data Analysis

Data analyses were conducted using SPSS 29.0 and the lavaan package in R (Rosseel, 2012). Descriptive statistics were computed to assess item-level distribution and detect potential anomalies. An exploratory factor analysis (EFA) using principal axis factoring with Promax rotation was conducted on Sample A to explore the underlying structure of the SSRPH. The number of factors to retain was determined using a combination of Kaiser’s criterion (eigenvalues > 1) and scree plot inspection (Zwick & Velicer, 1986).

Confirmatory factor analysis (CFA) was then conducted on Sample B to validate the factor structure identified in the EFA. Model fit was evaluated using the Comparative Fit Index (CFI), Tucker-Lewis Index (TLI), Root Mean Square Error of Approximation (RMSEA), and Standardized Root Mean Square Residual (SRMR), with thresholds of CFI and TLI ≥ 0.90, RMSEA ≤ 0.08, and SRMR ≤ 0.08 considered indicative of acceptable fit (Hu & Bentler, 1999).

Internal consistency was assessed using Cronbach’s alpha. Discriminant validity was examined by analyzing correlations between the SSRPH and the ATSPPH-SF scores.

## 3. Results

### 3.1. Exploratory Factor Analysis (EFA)

Exploratory factor analysis (EFA) was conducted using principal axis factoring with Promax rotation on Subsample A (*n* = 628). The dataset met the assumptions for factor analysis, with a Kaiser–Meyer–Olkin (KMO) value of 0.77, indicating adequate sampling adequacy, and Bartlett’s test of sphericity was significant, *χ*^2^(10) = 685.95, *p* < 0.001, confirming sufficient inter-item correlations.

The results of the EFA yielded a robust one-factor solution, with all five items retained. This single factor accounted for 56.52% of the total variance. All items demonstrated acceptable factor loadings (≥0.40), supporting a coherent unidimensional construct representing perceived public stigma toward psychological help-seeking. Table 1 presents an integrated summary of the factor loadings, inter-item correlations, and corrected item-total correlations, providing a comprehensive overview of each item’s psychometric performance.

### 3.2. Confirmatory Factor Analysis (CFA)

To verify the unidimensional factor structure identified in the EFA, a CFA was conducted on Subsample B (*n* = 629). The one-factor model demonstrated satisfactory overall fit: *χ*^2^(5) = 78.06, *p* < 0.001, CFI = 0.97, TLI = 0.95, and RMSEA = 0.096, 90% CI [0.061, 0.135]. While the RMSEA was marginally above the conventional threshold, the CFI and TLI values indicated a strong model fit.

**Figure 1:**
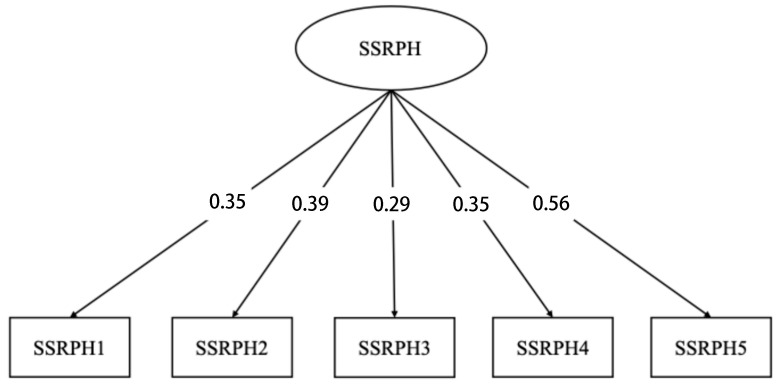
Standardized factor loading of the Stigma Scale for Receiving Psychological Help.

Standardized factor loadings ranged from 0.29 to 0.56 and were all statistically significant (*p* < 0.001), suggesting that each item contributed meaningfully to the latent construct. These findings support the structural validity of the Chinese version of SSRPH for use among Chinese law students. See Figure 1 for the path diagram of the CFA model.

### 3.3. Internal Consistency and Discriminant Validity

The internal consistency of the Chinese version of SSRPH was evaluated using Cronbach’s alpha. The scale demonstrated strong reliability, with Cronbach’s α = 0.82 in the total sample (N = 1257), suggesting good internal consistency among the five items.

To assess discriminant validity, bivariate correlations were computed between the SSRPH and the ATSPPH-SF. A significant negative correlation was observed (r = −0.37, *p* < 0.001), consistent with theoretical expectations and prior literature. This supports the scale’s ability to distinguish between stigma and attitudes toward help-seeking.

## 4. Discussion

### 4.1. Overview of Findings

The present study evaluated the psychometric properties of the Chinese version of the Stigma Scale for Receiving Psychological Help (SSRPH) among law students in mainland China. Results supported a unidimensional factor structure, with acceptable internal consistency and construct validity, consistent with prior validation studies of the SSRPH in other cultural contexts (e.g., Zavrou & Poulakis, 2017; Zhou et al., 2019). Confirmatory factor analysis demonstrated a good model fit, and the scale exhibited significant negative correlations with attitudes toward help-seeking, supporting its discriminant validity. These findings indicate that the Chinese version of SSRPH is a reliable and valid measure of perceived public stigma in a culturally and academically unique population.

While the confirmatory factor analysis demonstrated a strong overall fit (CFI = 0.97, TLI = 0.95), the RMSEA value (0.096) was marginally above conventional thresholds. This should be interpreted with caution, as RMSEA is known to overestimate model misfit in models with low degrees of freedom—a limitation well-documented in the structural equation modeling literature (Kenny et al., 2015). Given the theoretical grounding of the SSRPH and the parsimony of the one-factor structure, the model fit remains acceptable, particularly when considered alongside acceptable factor loadings, good internal consistency, and demonstrated discriminant validity.

In addition, several standardized factor loadings in the CFA were modest (ranging from 0.29 to 0.56), a pattern not uncommon in the validation of brief psychological scales addressing socially sensitive constructs. In such cases, lower loadings may still be psychometrically acceptable when items are theoretically coherent and contribute meaningfully to the construct (Kline, 2023; Brown, 2015). All items were statistically significant, and the scale demonstrated good internal consistency (Cronbach’s α = 0.82) and acceptable corrected item–total correlations (all > 0.30). For example, Item 5 demonstrated a corrected item–total correlation of 0.44, exceeding the recommended threshold for item retention (Costello & Osborne, 2005; Hair et al., 2009). Retaining all five items thus supports both the conceptual integrity and preliminary cross-cultural applicability of the SSRPH within the Chinese legal education context.

### 4.2. Theoretical Contributions

This study contributes to the growing literature on mental health stigma by validating a concise public stigma measure within a non-Western, high-pressure academic context. While previous studies have applied the SSRPH in various populations (e.g., Pinto et al., 2015; Zhou et al., 2019), this is the first to systematically assess its psychometric properties among Chinese law students—a group facing compounded challenges due to academic stress, professional expectations, and cultural barriers to help-seeking.

The findings reinforce the theoretical assumptions of the Theory of Planned Behavior, wherein perceived social norms, such as public stigma, play a critical role in shaping individuals’ intentions to seek psychological help. Specifically, the inverse relationship between stigma scores and help-seeking attitudes observed in this study reflects the critical role of subjective norms in shaping behavioral intentions. Furthermore, the data support Corrigan’s Social Cognitive Model of Stigma (Corrigan & Watson, 2002), as higher levels of perceived public stigma were associated with more negative help-seeking attitudes, suggesting an internalization of societal beliefs that discourage service use. This pattern underscores how social messages about mental illness may be cognitively processed and affect individual behavior. Lastly, the findings align with the Conservation of Resources theory, demonstrating that perceived stigma may be experienced as a threat to key psychosocial resources (e.g., social standing and professional identity), particularly salient in collectivist and competitive academic environments like legal education in China (Pinto et al., 2015). Together, these frameworks provide a coherent lens through which the observed relationships can be interpreted and contextualized.

### 4.3. Practical Implications

The validated SSRPH offers a contextually relevant and efficient tool for evaluating public stigma toward psychological help, which is a key proximal barrier to service use. Its unidimensional structure, acceptable reliability, and low respondent burden make it particularly well-suited for integration into large-scale campus surveys and mental health program evaluations (Komiya et al., 2000; Vogel et al., 2007). While it does not capture self-stigma, it serves as a practical screening measure to inform university-led efforts aimed at stigma reduction.

While the current study focused solely on the measurement of perceived public stigma, its findings provide a foundational basis for future research grounded in well-established psychological theories. By validating the Chinese version of the SSRPH within a high-pressure, culturally specific academic setting, this study contributes to understanding how stigma may operate as a socially normative barrier. Although we did not directly assess attitudes, behavioral intentions, or perceived resource threats, the present findings can inform future investigations that explicitly test theoretical models, such as the Theory of Planned Behavior and the Conservation of Resources framework.

Universities can integrate the SSRPH into screening protocols to better understand stigma dynamics within student populations (Yin et al., 2020). The validated SSRPH offers a practical and culturally appropriate instrument for screening and monitoring public stigma levels, thereby supporting the assessment and evaluation of anti-stigma efforts in university settings. Importantly, institutional policies should consider confidentiality, cultural sensitivity, and professional development needs when promoting mental health services among law students (Bibelhausen et al., 2015).

Furthermore, law schools may benefit from establishing proactive mental health literacy campaigns and embedding them into the legal curriculum (Fortner, 2022; Powell & Siller, 2024). These programs can frame psychological help-seeking as a strength rather than a weakness, counteracting normative concerns about “loss of face” or diminished professional image.

### 4.4. Limitations and Future Directions

Several limitations should be acknowledged. First, the sample was limited to undergraduate students from five universities in Shandong Province, potentially restricting generalizability to other regions, academic disciplines, or age groups. Future studies should extend validation efforts across more diverse educational, geographical, and cultural contexts.

Second, the reliance on self-report data may introduce social desirability bias, particularly given the sensitive nature of stigma. Multi-method approaches, including qualitative interviews or implicit association tests (IAT; Schimmack, 2021), could provide deeper insights into stigma-related constructs.

Third, the cross-sectional design does not permit conclusions about temporal stability or predictive validity. Longitudinal studies should examine whether SSRPH scores predict actual help-seeking behaviors and whether they are responsive to anti-stigma intervention over time.

Fourth, although the unidimensional model showed an acceptable fit based on theoretical considerations and multiple indices, the lack of comparison with alternative factor structures (e.g., bifactor or two-factor models) limits our ability to determine the most optimal representation. Future research should explore competing models and utilize fit indices, such as AIC and BIC, to evaluate structural robustness.

Lastly, cultural factors, such as family honor, relational obligations, or face concerns, which may be central to stigma expression in collectivist societies, were not directly assessed. Future research may enhance the SSRPH by incorporating culturally grounded items to better capture stigma within Confucian-influenced contexts.

## 5. Conclusions

The present study validates the Chinese version of the SSRPH for use among Chinese law students, offering a psychometrically sound and conceptually meaningful measure of public stigma. By confirming its unidimensional structure and establishing its associations with help-seeking attitudes, the study provides a foundation for further research and intervention in high-stress academic environments.

As public stigma continues to hinder access to mental health services among university students, especially in status-conscious and collectivist societies, reliable assessment tools are critical. This validated version of the SSRPH offers researchers, educators, and policymakers a practical means to evaluate stigma and to design informed, culturally appropriate strategies that support student well-being and professional development in legal education.

## Figures and Tables

**Table 1:** Inter-Item Correlations, Factor Loadings, and Corrected Item-Total Correlations for the SSRPH.

Item	1	2	3	4	5	Corrected Item-Total Correlation	Factor Loading
**SSRPH 1.** Seeing a psychologist for emotional or interpersonal problems carries social stigma.	1					0.59	0.69
**SSRPH 2.** It is a sign of personal weakness or inadequacy to see a psychologist for emotional or interpersonal problems.	0.47 ***	1				0.63	0.76
**SSRPH 3.** People will see a person in a less favorable way if they come to know that he/she has seen a psychologist.	0.45 ***	0.69 ***	1			0.69	0.74
**SSRPH 4.** It is advisable for a person to hide from people that he/she has seen a psychologist.	0.56 ***	0.40 ***	0.41 ***	1		0.78	0.63
**SSRPH 5.** People tend to like less those who are receiving professional psychological help.	0.38 ***	0.36 ***	0.32 ***	0.33 ***	1	0.45	0.49

***Notes.*** *** *p* < 0.001; SSRPH = Stigma Scale for Receiving Psychological Help.

## Data Availability

The data that support the findings of this study are available from the corresponding author upon reasonable request.

## Abbreviations

The following abbreviations are used in this manuscript:

EFA	Exploratory factor analysis
CFA	Confirmatory Factor Analysis
SSRPH	Stigma Scale for Receiving Psychological Help
ATSPPH-SF	Attitudes Toward Seeking Professional Psychological Help–Short Form
